# Improved tumor control with antiangiogenic therapy after treatment with gemcitabine and nab‐paclitaxel in pancreatic cancer

**DOI:** 10.1002/ctm2.398

**Published:** 2021-08-26

**Authors:** Zheng Zhang, Shunrong Ji, Qiangsheng Hu, Qifeng Zhuo, Wei Liu, Wenyan Xu, Wensheng Liu, Mengqi Liu, Zeng Ye, Guixiong Fan, Xiaowu Xu, Xianjun Yu, Yi Qin

**Affiliations:** ^1^ Department of Pancreatic Surgery Fudan University Shanghai Cancer Center Shanghai China; ^2^ Department of Oncology Shanghai Medical College Fudan University Shanghai China; ^3^ Shanghai Pancreatic Cancer Institute Shanghai China; ^4^ Pancreatic Cancer Institute Fudan University Shanghai China; ^5^ Department of Radiology Fudan University Shanghai Cancer Center Shanghai China

Dear Editor,

The regimen of nab‐paclitaxel and gemcitabine (AG) has been widely used as the first‐line chemotherapy for advanced pancreatic cancer; the prolonged survival time is still less than 2 months.^1^ Kim et al demonstrated that paclitaxel can induce vascular endothelial growth factor‐A (VEGF) expression which could facilitate the survival of neoplastic and tumor cells, thus protecting both endothelial and stroma cells from cytotoxic death while promoting angiogenesis.^2^, ^3^, ^4^

To explore the neovascularization in patients with pancreatic ductal adenocarcinoma (PDAC) treated with AG or gemcitabine alone, we first examined the *K^trans^* value in the tumor by Dynamic contrast enhancement magnetic resonance imaging (DCE‐MRI). Patients with pancreatic cancer treated with the three‐cycle AG regimen had significantly elevated *K^trans^* (Figures 1A and 1B), but patients who received gemcitabine monotherapy showed only a slight change in *K^trans^* (Figure S1A). Clinical characteristics of the patients in Figures 1 and S1 are summarized in Tables S1, S2, and S3. Similarly, extravascular extracellular space volume ratio (V_e_), the rate constant (K_ep_), and initial area under the curve taken up to 60 s (iAUC60), were elevated in the AG group (Figures 1C–1E), but not in the gemcitabine group (Figures S1B‐S1D). Using a protein array consisting of 440 human angiogenic factors, growth factors, chemokines and inflammatory factors, we found that the VEGF, was significantly elevated in the AG group using human cytokine array (Figure 1F). Moreover, the level of VEGF did not change significantly in the gemcitabine monotherapy group (Figure S1E). We confirmed by enzyme linked immunosorbent assay that there was a significantly high VEGF level in the AG group, but not in the gemcitabine group (Figures 1G and S1F). Moreover, the IF results revealed that compared with the patients who obtained neoadjuvant therapy with gemcitabine alone and those who did not receive neoadjuvant therapy, the patients who obtained neoadjuvant therapy with AG had increased significantly blood vessels (Figures 1H and 1I), thicker blood vessels (Figure S1G), and decreased significantly stroma in tumor tissue (Figures 1H and 1J).

**FIGURE 1:**
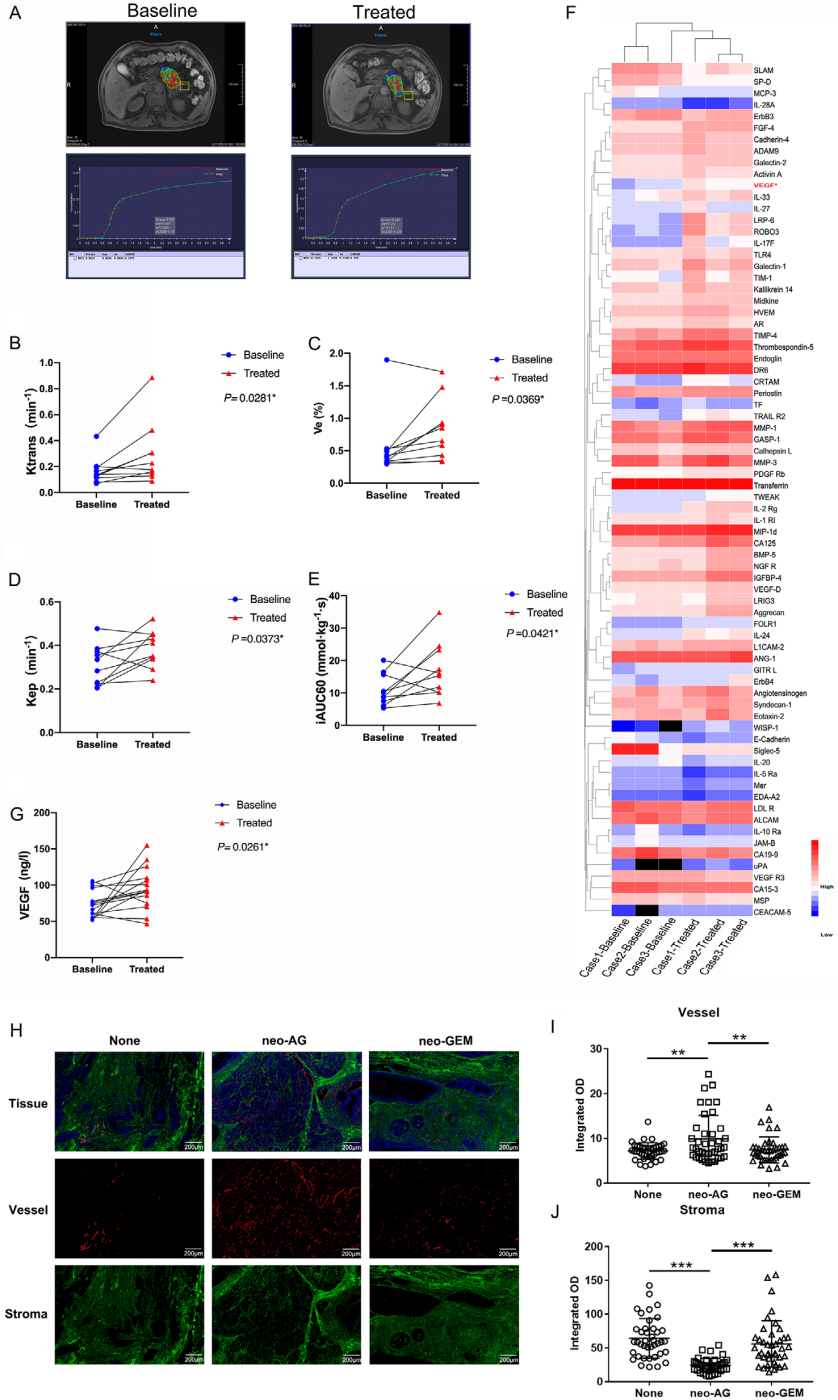
Correlation between gemcitabine‐based treatment and either *K^trans^* or VEGF in the AG group. (A) Representative DCE‐MRI image of patient with pancreatic cancer with altered *K^trans^*. (B‐E) Statistical analysis of the *K^trans^*, V_e_, K_ep_, and iAUC60, respectively, in the AG group. (F) Human cytokine array analysis of the effect of VEGF stimulation on the expression of putative genes related to gemcitabine sensitivity. (G) Statistical analysis of the difference in VEGF expression in the AG group. (H) Representative IF image of patients treated by neoadjuvant therapy. (I and J) Statistical analysis of the integrated OD of vessel and stroma. The baseline is referring to the patient before therapy administration

Interesting, VEGF promotes gemcitabine resistance in pancreatic cancer cells (Figures 2A and 2B). Next, we performed transcriptome sequencing and identified several signaling pathways that were changed after VEGF stimulation (Figure 2C). Among the differentially expressed genes, RRM1 is an important molecule for gemcitabine efficacy, and there is a directly correlation between RRM1 and gemcitabine resistance.^5^, ^6^ The mRNA and protein levels of RRM1 increased following VEGF stimulation (Figures 2D and 2E). Moreover, our sequencing data show that VEGF can upregulate c‐Myc expression, and the results were confirmed by q‐PCR and western blot (Figures 2F and 2G). Additionally, a positive correlation was observed between VEGF and RRM1 (Figures 2H and 2I). Furthermore, Western Blot results showed that the more sensitive the cells were to gemcitabine (corresponding to the lower IC50), the lower the expression levels of RRM1 and VEGF were (Figure 2J). Taken together, these results strongly indicate that RRM1 is a potential VEGF target in PDAC.

**FIGURE 2:**
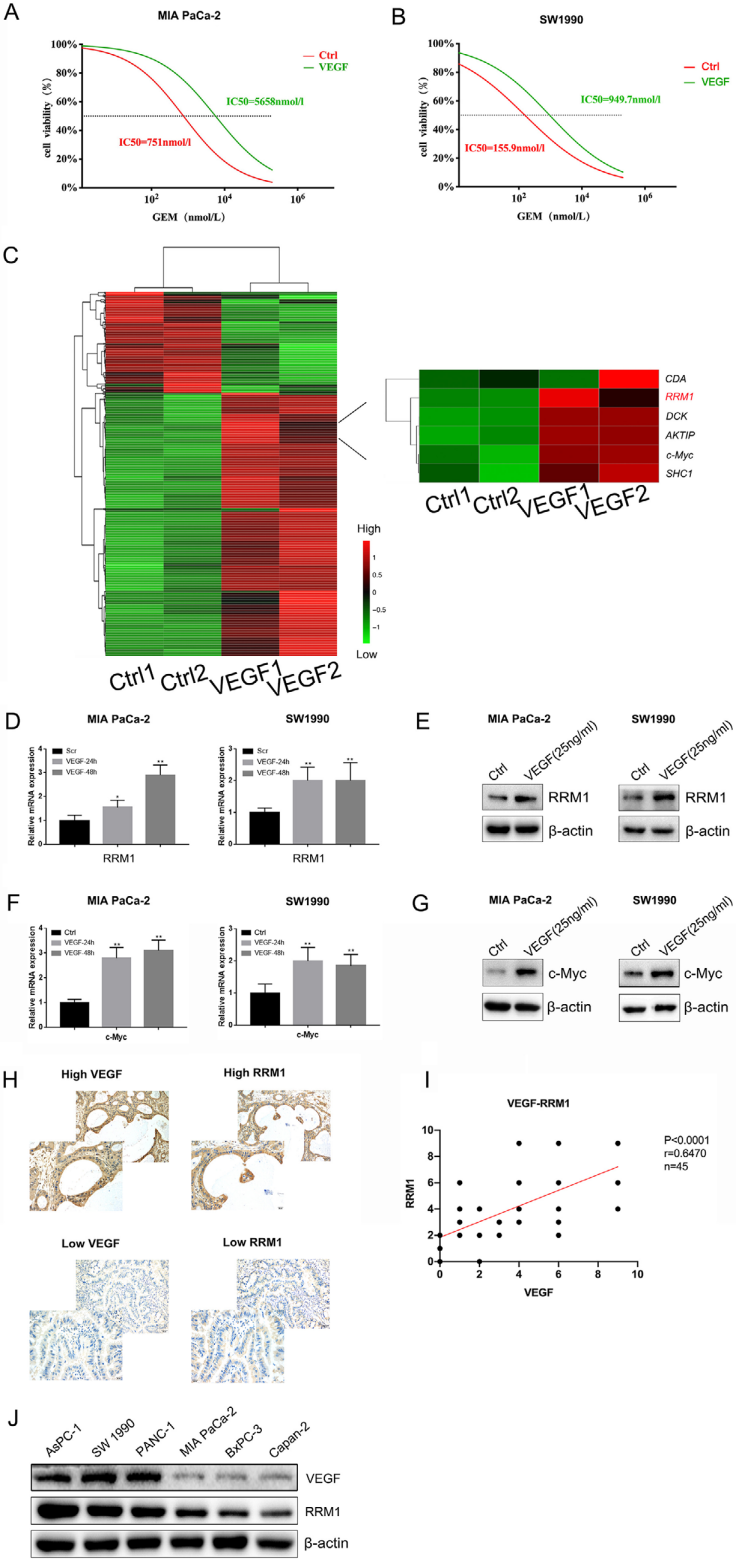
Screening for VEGF effector proteins related to gemcitabine sensitivity. (A and B) Gemcitabine cytotoxicity is negated by VEGF in MIA PaCa‐2 and SW 1990 cells. (C) Transcriptome sequencing revealed that VEGF stimulation upregulated RRM1 expression. (D and E) VEGF increased the RRM1 mRNA and protein levels. (F and G) The mRNA and protein levels of c‐Myc are elevated upon VEGF stimulation. (H and I) VEGF and RRM1 expression suggested a positive correlation in clinical samples from patients with PDAC. (J) The protein levels of VEGF and RRM1 in six kinds of pancreatic cancer cell lines

A previous report stated that the levels of RRM1 were significantly reduced in myc‐depleted cells.^7^ We first examined RRM1 mRNA (Figures 3A and 3B) and protein (Figures 3C and 3D) expression in pancreatic cancer cells treated by JQ‐1 or transfected with siRNA. We found two putative c‐Myc‐binding E‐box elements in the promoter region of RRM1 (Figure 3E). Next, the dual‐luciferase assay indicated that cotransfection with c‐Myc promoted RRM1 promoter activity in a dose‐dependent manner (Figure 3F). Moreover, ChIP results demonstrated that c‐Myc occupied the E‐boxes in the RRM1 promoter region (Figure 3G). These data indicate that c‐Myc could promote RRM1 transcription. To further confirm the role of c‐Myc in the effect of VEGF on RRM1, we targeted both VEGF and c‐Myc in cells. As expected, RRM1 mRNA (Figures S2A and S2B) and protein (Figures S2C and S2D) levels were significantly lowered, while c‐Myc was silenced by, JQ‐1 or siRNA in pancreatic cancer cells, even when VEGF was stimulated. Finally, we silenced c‐Myc in VEGF‐stimulated MIA PaCa‐2 and SW1990 cells, and found that c‐Myc suppression or knockdown could reverse the effects of VEGF on the IC50 values of gemcitabine of pancreatic cancer cells (Figures S2E and S2F).

**FIGURE 3:**
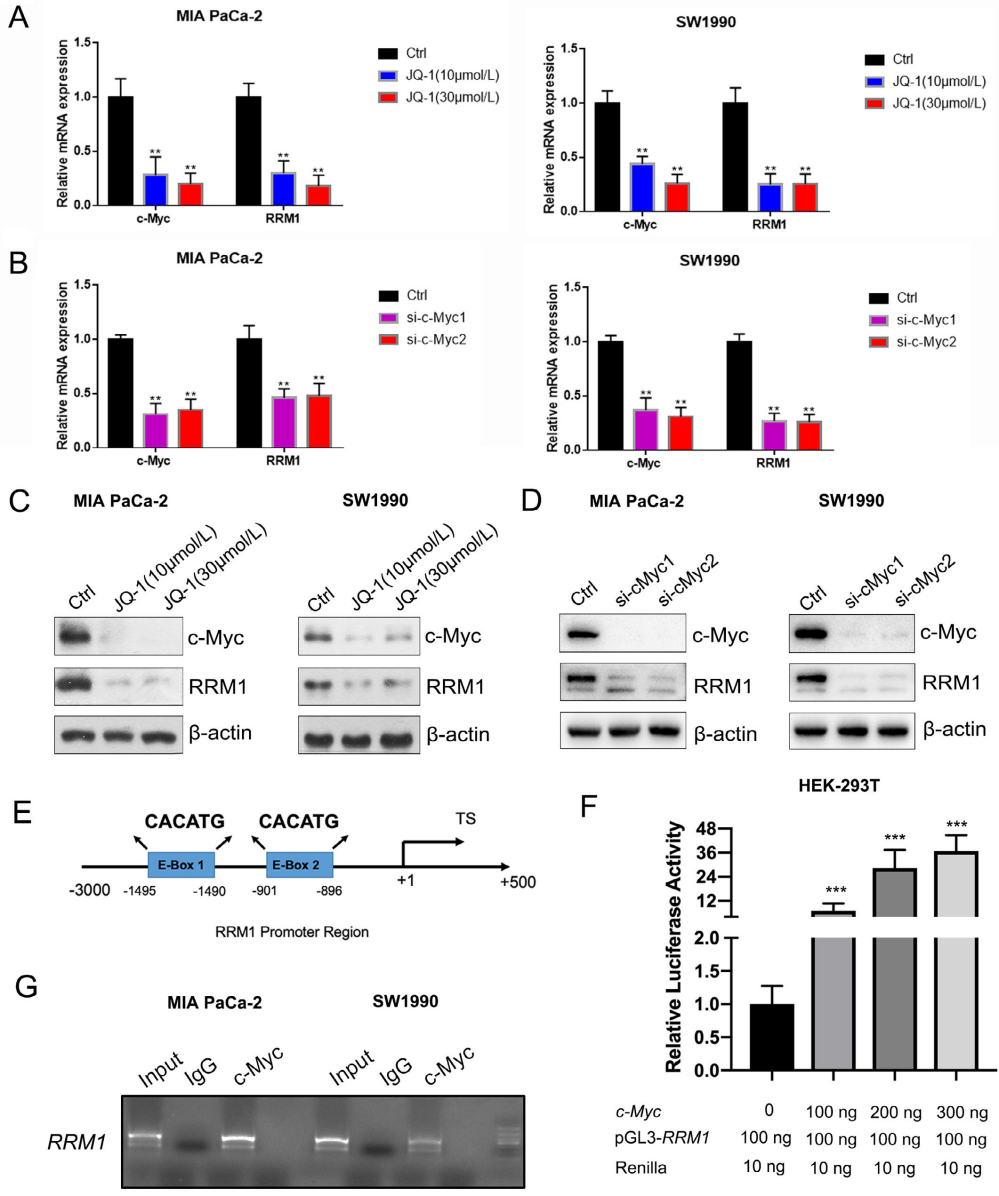
VEGF upregulates RRM1 via c‐Myc. (A‐D) JQ‐1‐ or siRNA‐mediated blockage of c‐Myc activity/expression led to a decrease in RRM1 mRNA and protein levels. (E) Position of the c‐Myc binding sites in the RRM1 promoter. (F) Relative RRM1 promoter activity in HEK‐293T cells cotransfected with the RRM1 promoter and a c‐Myc‐expression plasmid. (G) c‐Myc occupies the E‐box of the RRM1 promoter region as measured by ChIP

One PDX model of human PDAC was also established. AG showed a stronger antitumor effect than gemcitabine alone (Figure 4A). More importantly, salvage therapy combining bevacizumab with AG further reduced the tumor size compared with AG treatment alone (Figure 4B). The IF results showed compared with the mice treated by gemcitabine alone or 0.9% normal saline, those treated by AG or ABX had increased significantly blood vessels (Figures 4C, 4D, and 4F), thicker blood vessels (Figure 4E), and decreased significantly stroma in tumor tissue (Figures 4C, 4D, and 4G). As expected, the serum VEGF of the mice in the AG and paclitaxel groups was elevated significantly at day 28, but not in the gemcitabine group or control group (Figures 4H–4K).

**FIGURE 4:**
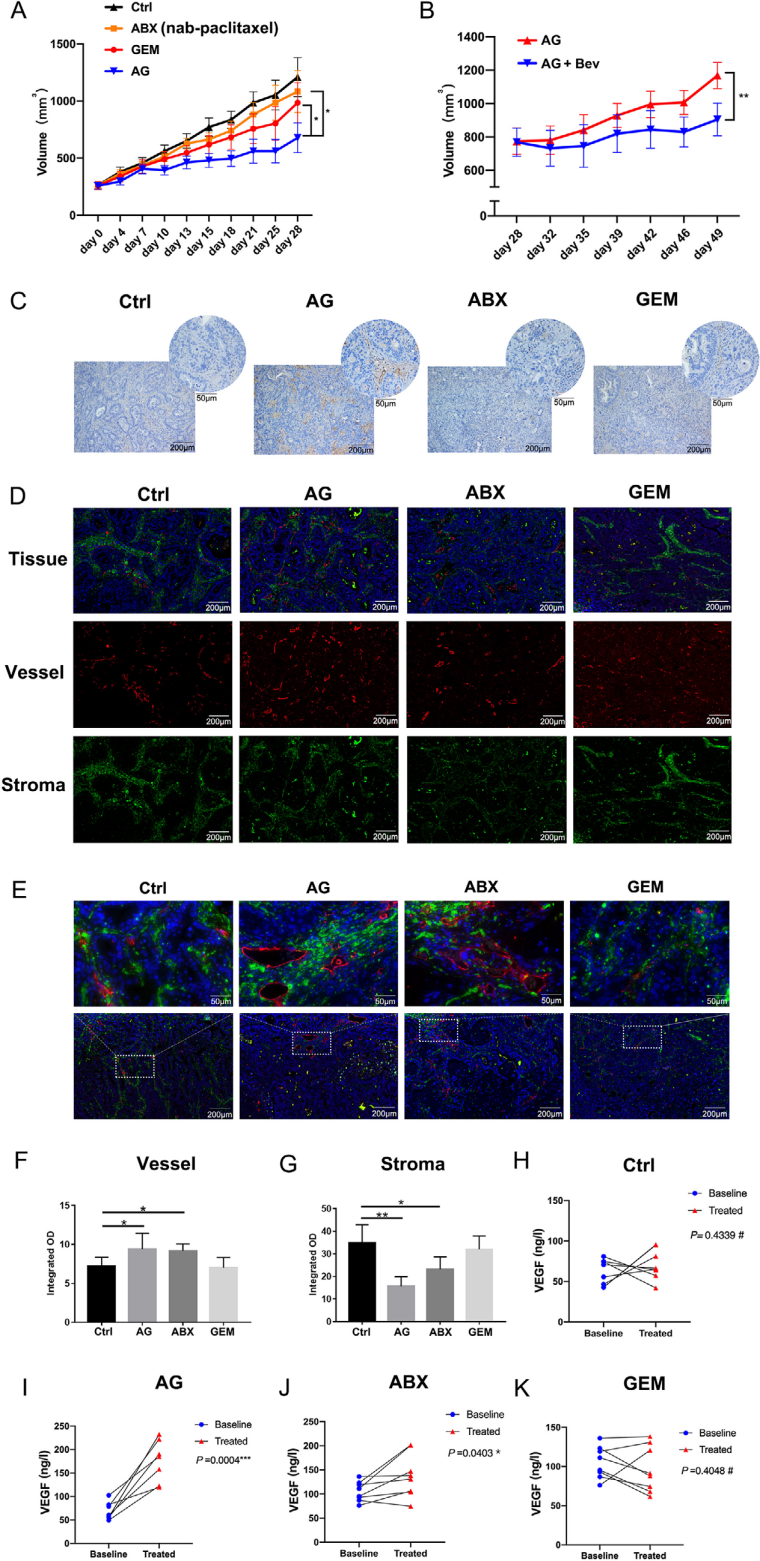
The addition of bevacizumab enhances the efficacy of AG in a PDX model. (A) The cytotoxicity of AG and gemcitabine monotherapy in mice. (B) The effect of AG plus bevacizumab compared with AG. (C) Representative IHC images of mice treated by 0.9% NS or chemotherapy. (D and E) Representative IF images of mice treated by 0.9% NS or chemotherapy. (F and G) Statistical analysis of the integrated OD of vessel and stroma. (H‐K) Statistical analysis of the difference in VEGF of mouse among the different therapy groups. Baseline is referring to the basic value of each index of each individual before treatment

A 59‐year‐old male patient was diagnosed with PDAC confirmed by pathology. The CT and DCE‐MRI showed pancreatic body tumor and liver metastasis. After two cycles of AG treatment, both the primary and metastatic lesions were stable. In addition, he described that his back pain was significantly relieved. After two additional cycles of AG treatment, the primary tumor shrunk to some extent, while the liver metastatic tumor remained stable. However, new nodules (red arrows) in the right lung emerged (Figures S3A and S3B). Although the nodule was too small (∼1 cm) to be biopsied, pathologists and radiologists still evaluated the nodule as a metastasis by comprehensive assessment. Because peripheral blood VEGF was elevated (Figure S3C), the AG regimen plus bevacizumab was administered to the patient, which is safe and well‐tolerated.^8^ After two cycles of treatment with AG plus bevacizumab, the primary lesions were smaller than before, and some of the nodules in the right upper lung disappeared. Bevacizumab showed good synergistic effects with AG after disease progression in our patient with pancreatic cancer, although he eventually died due to cancer progression.

Overall, these observations suggest that there were more blood vessels and less stroma in the tumor tissue after 2–4 cycles AG treatment (Figure S3D), and that the addition of bevacizumab to AG exerted a benefit in pancreatic cancer.

## CONFLICT OF INTEREST

The authors have declared no conflict of interest.

## AUTHOR CONTRIBUTIONS

This study was conceived by Yi Qin, Xiaowu Xu, and Xianjun Yu. Zheng Zhang and Shunrong Ji designed the study. Zheng Zhang and Qifeng Zhuo performed the experiments. Zheng Zhang, Qiangsheng Hu, Wei Liu, and Yi Qin analyzed and interpreted the data. Wei Liu, Wenyan Xu, Guixiong Fan, Mengqi Liu, and Zeng Ye reviewed the manuscript. Zheng Zhang wrote the paper with comments from all authors. All authors read and approved the final manuscript.

## ETHICS APPROVAL AND CONSENT TO PARTICIPATE

We have gained the informed consent of patients and approval from the Clinical Research Ethics Committee of Fudan University Shanghai Cancer Center. The reported patient gave consent for the publication of his case. All animal experimental procedures were performed in accordance with the protocols approved by the Institutional Animal Care and Research Advisory Committee of Fudan University Shanghai Cancer Center.

## DATA AVAILABILITY STATEMENT

All data generated or analyzed during this study are included in this published article and its supplementary information files or from the corresponding author upon reasonable request.

## Supporting information

**Figure S1** Statistical analysis of the correlation between the gemcitabine‐based regimen and *K^trans^* or VEGF in the gemcitabine group. (A‐D) Statistical analysis of *K^trans^*, V_e_, K_ep_, and iAUC60 in the gemcitabine group. (E) Human cytokine array analysis of the effect of VEGF stimulation on the expression of putative genes involved in gemcitabine sensitivity in the gemcitabine group. (F) Statistical analysis of the difference in VEGF in the gemcitabine group. (G) Representative IF image of patients treated by neoadjuvant therapy. The baseline is referring to the patient before therapy administration.

**Figure S2** Silencing c‐Myc reverses the effect of VEGF on RRM1. (A‐D) The mRNA and protein levels of RRM1 were lowered by silencing c‐Myc, even with VEGF stimulation. (E and F) c‐Myc suppression or knockdown reversed the effects of VEGF on the IC50 values of gemcitabine of pancreatic cancer cells.

**Figure S3** A real‐world case of a patient with pancreatic cancer treated with AG plus bevacizumab after progression in response to AG. (A and B) Treatment and disease progression for the presented case. (C) Changes in the VEGF levels of the patient during chemotherapy. (D) The graphical representation summarizes the pancreatic cancer tissues before and after AG treatment.

Table S1. Clinicopathological features and correlation of *K^trans^* in PDAC.
Table S2. Clinicopathological features and correlation of serum VEGF in PDAC.
Table S3. Clinicopathological features and correlation of VEGF expression in PDAC.
