# Supplementary figures and images for: Improved tumor control with antiangiogenic therapy after treatment with gemcitabine and nab‐paclitaxel in pancreatic cancer
Source: Clin Transl Med. 2021 Aug 26;11(8):e398. doi: 10.1002/ctm2.398 (PMC8387784; doi:10.1002/ctm2.398)

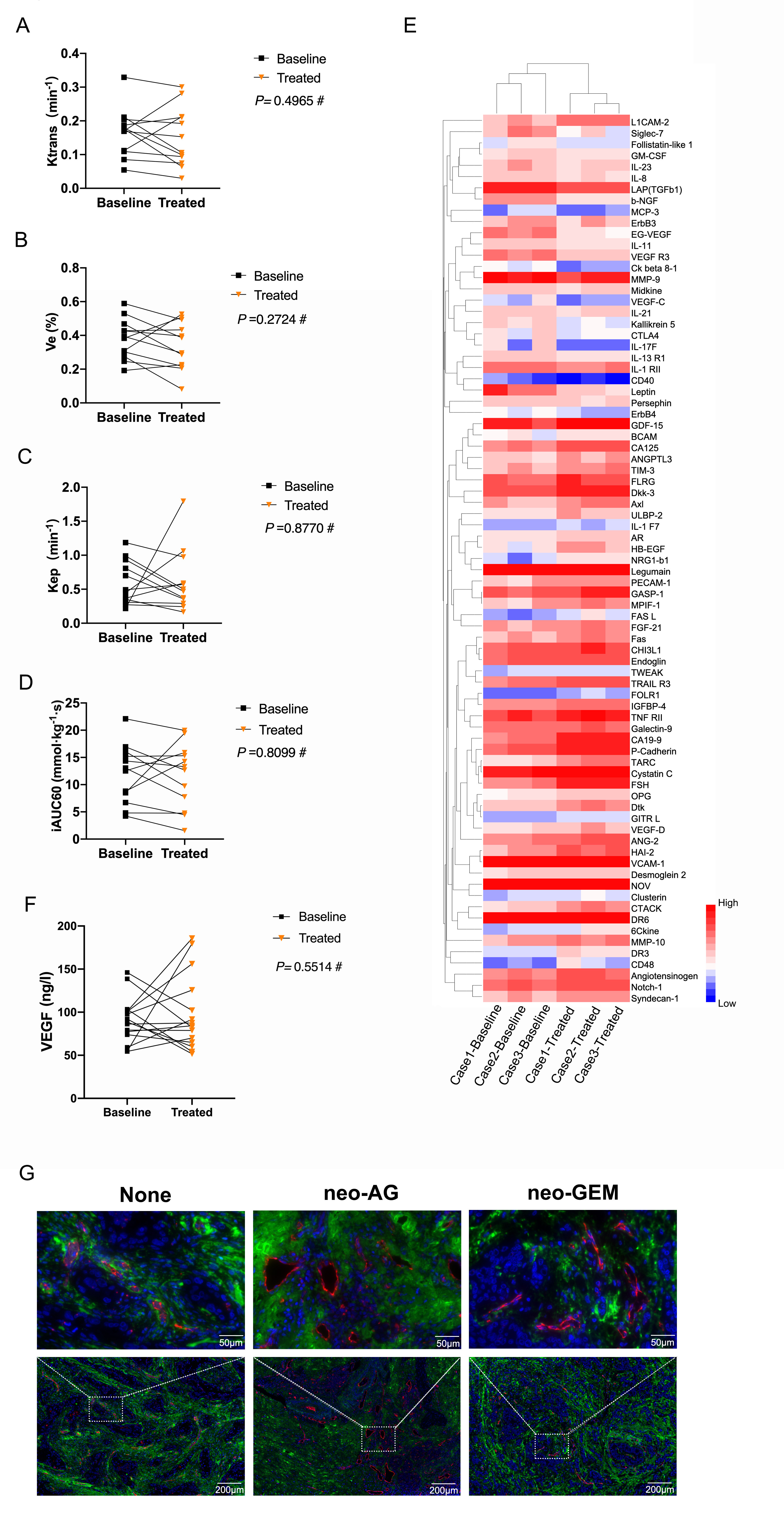

Supplement: Supplementary file 1 — Figure S1 Statistical analysis of the correlation between the gemcitabine‐based regimen and Ktrans or VEGF in the gemcitabine group. (A‐D) Statistical analysis of Ktrans , Ve, Kep, and iAUC60 in the gemcitabine group. (E) Human cytokine array analysis of the effect of VEGF stimulation on the expression of putative genes involved in gemcitabine sensitivity in the gemcitabine group. (F) Statistical analysis of the difference in VEGF in the gemcitabine group. (G) Representative IF image of patients treated by neoadjuvant therapy. The baseline is referring to the patient before therapy administration. [file CTM2-11-e398-s001.jpg]

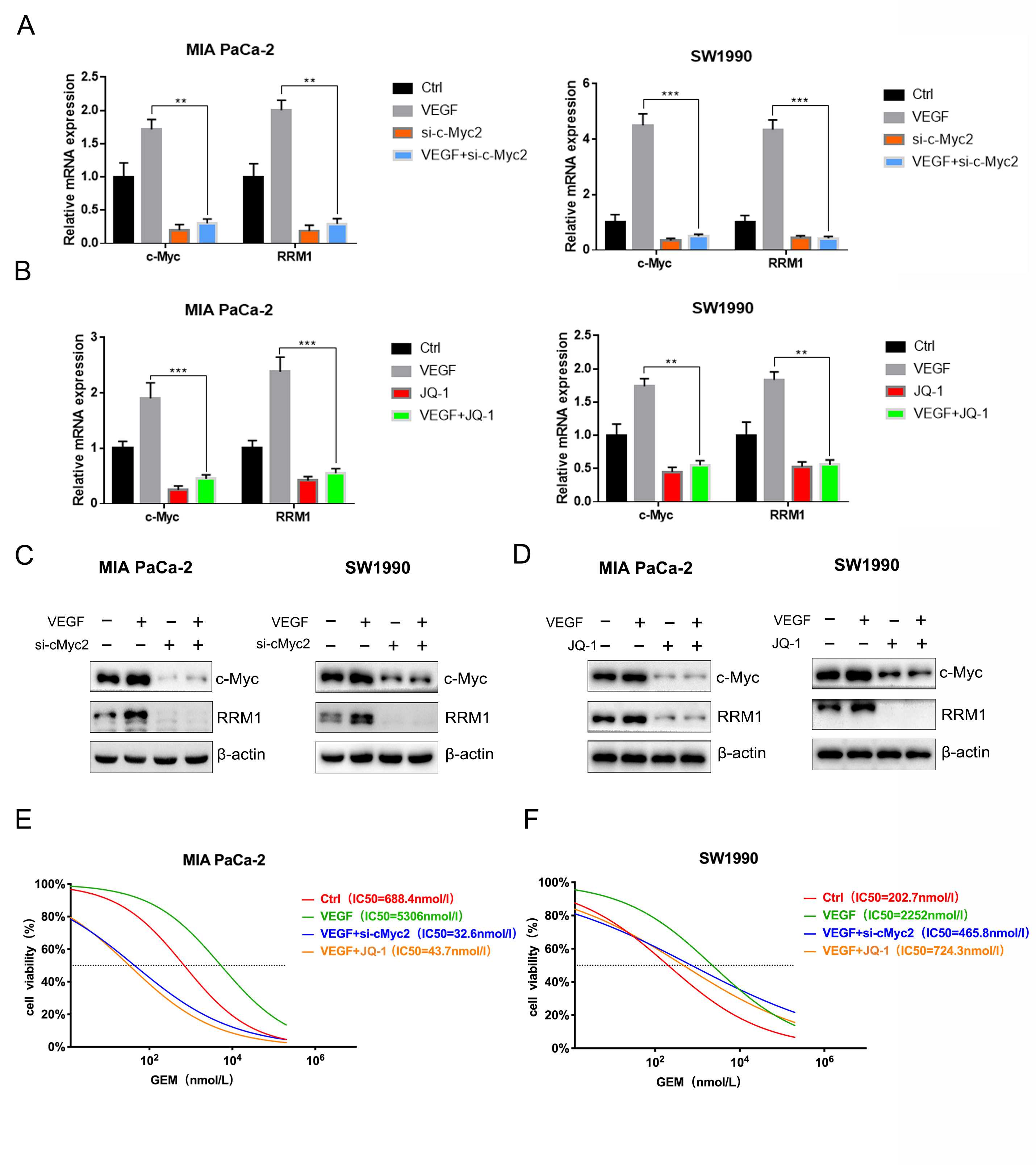

Supplement: Supplementary file 2 — Figure S2 Silencing c‐Myc reverses the effect of VEGF on RRM1. (A‐D) The mRNA and protein levels of RRM1 were lowered by silencing c‐Myc, even with VEGF stimulation. (E and F) c‐Myc suppression or knockdown reversed the effects of VEGF on the IC50 values of gemcitabine of pancreatic cancer cells. [file CTM2-11-e398-s004.jpg]

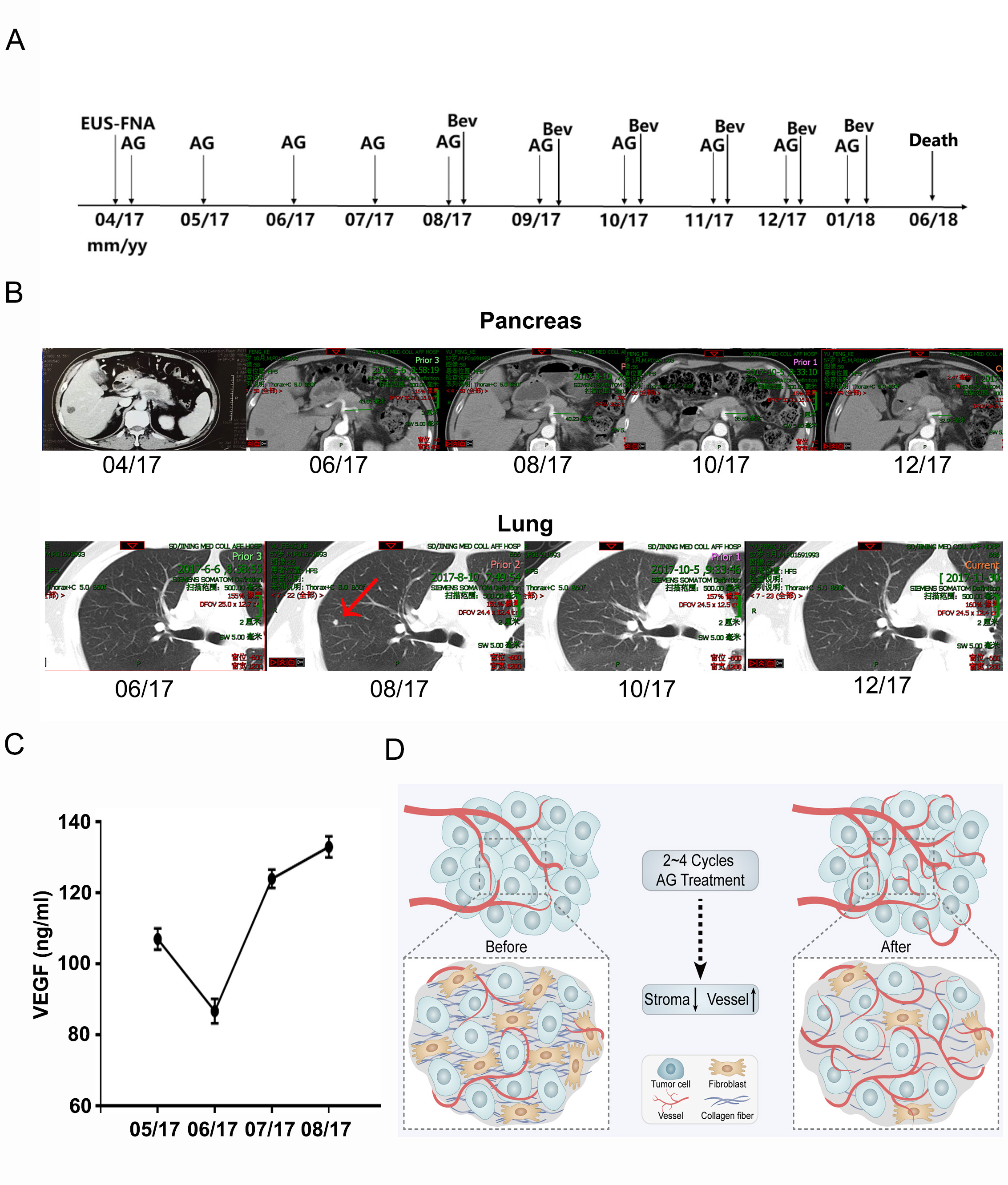

Supplement: Supplementary file 3 — Figure S3 A real‐world case of a patient with pancreatic cancer treated with AG plus bevacizumab after progression in response to AG. (A and B) Treatment and disease progression for the presented case. (C) Changes in the VEGF levels of the patient during chemotherapy. (D) The graphical representation summarizes the pancreatic cancer tissues before and after AG treatment. [file CTM2-11-e398-s002.jpg]
