# Supplementary material for: Improved tumor control with antiangiogenic therapy after treatment with gemcitabine and nab‐paclitaxel in pancreatic cancer
Source: Clin Transl Med. 2021 Aug 26;11(8):e398. doi: 10.1002/ctm2.398 (PMC8387784; doi:10.1002/ctm2.398)
Supplement: Supplementary file 4 — Table S1. Clinicopathological features and correlation of Ktrans in PDAC. Table S2. Clinicopathological features and correlation of serum VEGF in PDAC. Table S3. Clinicopathological features and correlation of VEGF expression in PDAC. [file CTM2-11-e398-s003.docx]

**Table S1. Clinicopathological features and correlation of *K^trans^* in PDAC.**

|  |  | ***K^trans^* -Low** | ***K^trans^* -High** |  |
| --- | --- | --- | --- | --- |
| **Characteristics** | **No.** | **10** | **12** | ***P* Value** |
| **Age (y)** |  |  |  | 0.666 |
| **<60** | 9 | 5 | 4 |  |
| **≥60** | 13 | 5 | 8 |  |
| **Gender** |  |  |  | 0.646 |
| **Female** | 6 | 2 | 4 |  |
| **Male** | 16 | 8 | 8 |  |
| **Tumor size (cm)** |  |  |  | 0.652 |
| **<4.0** | 7 | 4 | 3 |  |
| **≥4.0** | 15 | 6 | 9 |  |
| **Lymph node status** |  |  |  | 0.378 |
| **Negative** | 8 | 5 | 3 |  |
| **Positive** | 14 | 5 | 9 |  |
| **Pathological N** |  |  |  | 0.293 |
| **N0** | 4 | 3 | 1 |  |
| **N1** | 18 | 7 | 11 |  |
| **Pathological M** |  |  |  | 0.074 |
| **M0** | 15 | 9 | 6 |  |
| **M1** | 7 | 1 | 6 |  |
| **Pathological T** |  |  |  | 0.594 |
| **T1/T2** | 4 | 1 | 3 |  |
| **T3/T4** | 18 | 9 | 9 |  |
| **Stage** |  |  |  | 0.571 |
| **I-IIA** | 3 | 2 | 1 |  |
| **IIB-VI** | 19 | 8 | 11 |  |

P values were derived with Chi-square test or Fisher‘s exact test; all statistical tests are two sided.

**Table S2. Clinicopathological features and correlation of serum VEGF in PDAC.**

|  |  | **VEGF-Low** | **VEGF-High** |  |
| --- | --- | --- | --- | --- |
| **Characteristics** | **No.** | **15** | **15** | ***P* Value** |
| **Age (y)** |  |  |  | 0.715 |
| **<60** | 14 | 6 | 8 |  |
| **≥60** | 16 | 9 | 7 |  |
| **Gender** |  |  |  | 0.71 |
| **Female** | 12 | 5 | 7 |  |
| **Male** | 18 | 10 | 8 |  |
| **Tumor size (cm)** |  |  |  | 0.45 |
| **<4.0** | 11 | 4 | 7 |  |
| **≥4.0** | 19 | 11 | 8 |  |
| **Lymph node status** |  |  |  | 0.682 |
| **Negative** | 8 | 3 | 5 |  |
| **Positive** | 22 | 12 | 10 |  |
| **Pathological N** |  |  |  | 0.651 |
| **N0** | 6 | 4 | 2 |  |
| **N1** | 24 | 11 | 13 |  |
| **Pathological M** |  |  |  | 0.71 |
| **M0** | 18 | 8 | 10 |  |
| **M1** | 12 | 7 | 5 |  |
| **Pathological T** |  |  |  | 0.33 |
| **T1/T2** | 5 | 4 | 1 |  |
| **T3/T4** | 25 | 11 | 14 |  |
| **Stage** |  |  |  | 0.598 |
| **I-IIA** | 4 | 1 | 3 |  |
| **IIB-VI** | 26 | 14 | 12 |  |

P values were derived with Chi-square test or Fisher‘s exact test; all statistical tests are two sided.

**Table S3. Clinicopathological features and correlation of VEGF expression in PDAC.**

|  |  | **VEGF-Low** | **VEGF-High** |  |
| --- | --- | --- | --- | --- |
| **Characteristics** | **No.** | **31** | **14** | ***P* Value** |
| **Age (y)** |  |  |  | 0.731 |
| **<60** | 24 | 16 | 8 |  |
| **≥60** | 21 | 15 | 6 |  |
| **Gender** |  |  |  | 0.988 |
| **Female** | 16 | 11 | 5 |  |
| **Male** | 29 | 20 | 9 |  |
| **Tumor size (cm)** |  |  |  | 0.497 |
| **<4.0** | 32 | 23 | 9 |  |
| **≥4.0** | 13 | 8 | 5 |  |
| **Lymph node status** |  |  |  | 0.356 |
| **Negative** | 33 | 24 | 9 |  |
| **Positive** | 12 | 7 | 5 |  |
| **Pathological N** |  |  |  | 0.115 |
| **N0** | 27 | 21 | 6 |  |
| **N1** | 18 | 10 | 8 |  |
| **Pathological M** |  |  |  | 0.464 |
| **M0** | 42 | 30 | 12 |  |
| **M1** | 3 | 1 | 2 |  |
| **Pathological T** |  |  |  | 0.053 |
| **T1/T2** | 11 | 5 | 6 |  |
| **T3/T4** | 34 | 26 | 8 |  |
| **Stage** |  |  |  | 0.356 |
| **I-IIA** | 12 | 7 | 5 |  |
| **IIB-VI** | 33 | 24 | 9 |  |

P values were derived with Chi-square test or Fisher‘s exact test; all statistical tests are two sided.
